# Advancing AMD Detection: Dataset Design and Deep Learning Optimization for Unconstrained Retinal Images

**DOI:** 10.3390/vision10020028

**Published:** 2026-05-14

**Authors:** Hala Nafie Fathee, Reyhan Babayev, Shaaban Sahmoud, Nazim Ağaoğlu

**Affiliations:** 1 College of Physical Education and Sport Sciences, University of Mosul, Mosul 41002, Iraq; hala.fathee@uomosul.edu.iq; 2 Istanbul Göz Hospital, 34180 Istanbul, Türkiye; reyhamid@yahoo.com; 3 Computer Engineering Department, Fatih Sultan Mehmet Vakif University, 34421 Istanbul, Türkiye; 4 Department of Mathematics, Istinye University, 34396 Istanbul, Türkiye; nazim.agaoglu@istinye.edu.tr

**Keywords:** AMD detection, unconstrained retinal images, age-related macular degeneration, AMD dataset, deep learning

## Abstract

Age-related macular degeneration (AMD) is one of the leading causes of vision impairment worldwide, making early and accurate detection essential for effective clinical intervention. Recent advances in deep learning have demonstrated promising results in automated retinal image analysis; however, most existing approaches rely on datasets acquired under controlled conditions, limiting their generalizability to real-world clinical environments. In this paper, we propose a novel AMD dataset designed to simulate unconstrained imaging conditions, by incorporating noise, luminance variations, and device-related artifacts commonly encountered during retinal scan acquisition. Using this dataset, we conduct a comprehensive comparative evaluation of six widely adopted deep learning architectures: VGG16, VGG19, InceptionV3, MobileNetV2, ResNet50, and DenseNet. Experimental results indicate notable performance variations across models, highlighting the impact of architectural design on robustness to image degradation. Among the evaluated approaches, VGG16 achieved the best overall performance. By further optimizing this architecture through targeted training and fine-tuning strategies, the proposed system reached an accuracy of 88% in AMD detection. These findings demonstrate the effectiveness of the optimized VGG16 model and underline the importance of realistic datasets for developing reliable deep learning-based diagnostic tools for practical clinical settings.

## 1. Introduction

Medical imaging plays a crucial role in the diagnosis and treatment of various diseases and conditions. However, the tools currently available for analyzing and interpreting medical images often fall short, leading to inconsistencies in diagnoses and treatment decisions. This issue is particularly significant in regions with limited access to specialized medical equipment and well-trained healthcare professionals.

One critical challenge in medical image processing is the variability in image quality. Medical images acquired through modalities such as X-rays, CT scans, and MRI often exhibit variations due to factors such as patient movement, differences in imaging equipment, and acquisition parameters. This variability can hinder accurate processing and interpretation, thereby increasing the likelihood of misdiagnosis or missed abnormalities [1,2,3,4]. Moreover, the vast volume of data generated by medical imaging presents another challenge. Healthcare professionals often struggle to effectively analyze and interpret these data, underscoring the need for fast and efficient image processing algorithms. Especially in real-time clinical settings, quick and accurate analysis of medical images is critical for patient diagnosis and treatment. However, many current methods are computationally intensive and struggle to process large datasets in real time. The development of more robust and efficient algorithms is essential to overcome these limitations and improve clinical outcomes [5,6,7,8]. Advanced CNN architectures such as those described in [8] play a critical role in enhancing segmentation accuracy by leveraging multi-scale feature extraction.

Medical image processing also faces other significant issues, including image quality, as low-quality images can impede accurate diagnosis, potentially leading to incorrect treatments and endangering patient health [9,10]. Image quality standardization, as highlighted in [10], ensures consistency and improves AI model reliability. Data privacy is another concern, given the increasing reliance on electronic medical records and cloud-based storage, which raises issues regarding the privacy and security of patient data [11,12]. Additionally, data standardization remains a challenge due to variations in image formats across different devices and software, complicating data sharing and analysis between healthcare providers [13,14,15]. Effective data management is crucial, as the vast amounts of data generated by imaging devices, particularly in terms of storage and retrieval, pose significant challenges [16]. Algorithm accuracy is also essential for reliable results, as false positives or negatives can lead to serious consequences [17]. Furthermore, the cost of medical image processing, which often requires expensive equipment, specialized software, and trained professionals, may limit its accessibility [18].

To address these challenges, collaborative efforts involving medical professionals, software developers, and researchers are necessary. The integration of innovative technologies such as machine learning and artificial intelligence holds great promise for advancing the field. These technologies can improve the accuracy and efficiency of image analysis, streamline data management, and enable real-time decision-making in clinical environments [19,20,21,22]. Ensemble learning methods, as discussed in [22], further enhance model reliability by combining predictions from multiple algorithms.

The eye, as a complex and delicate organ, plays an essential role in providing a sense of sight. Unfortunately, it is highly susceptible to a wide array of diseases that can result in vision loss, eye pain, and other debilitating symptoms. The causes of eye diseases are diverse and include genetic predisposition, aging, injuries, and underlying health conditions. Common conditions such as cataracts, glaucoma, diabetic retinopathy, and dry eye syndrome affect millions worldwide. Among these, Age-Related Macular Degeneration (AMD) stands out as one of the leading causes of vision loss in older adults. AMD is a progressive condition that primarily impacts the macula, the central region of the retina responsible for sharp central vision. This vision is critical for daily activities such as reading, driving, and recognizing faces. Although the exact etiology of AMD remains unknown, risk factors such as advancing age, genetics, and certain lifestyle factors, including smoking and poor dietary habits, have been identified.

Symptoms of AMD typically begin with subtle changes, such as blurred or distorted central vision and difficulty performing tasks requiring fine detail. As the disease progresses, patients may experience significant distortion, blind spots, or complete loss of central vision, profoundly affecting their independence and quality of life.

In this study, we present a comprehensive framework for automated age-related macular degeneration (AMD) detection under unconstrained imaging conditions. Our primary contribution is the introduction of a novel AMD dataset specifically designed to realistically mimic real-world clinical environments, where imaging artifacts such as noise, luminance variations, blur, and device-related inconsistencies frequently occur during scan acquisition. While several publicly available retinal datasets exist, such as ORIGA, DRIVE, STARE, and MESSIDOR, these benchmarks are predominantly captured under standardized, controlled acquisition protocols, limiting their ability to represent the variability encountered in routine clinical screening. In contrast, the proposed dataset is intentionally constructed to include heterogeneous imaging conditions, varying patient demographics, and diverse device configurations, thereby bridging the critical gap between laboratory-grade benchmarks and real-world deployability. Moreover, unlike existing AMD-specific datasets, which are often limited in scale, class diversity, or imaging variability, our dataset encompasses a broader spectrum of disease presentations alongside non-AMD cases, enabling more robust and generalizable model training. Furthermore, we conduct an extensive comparative evaluation of six widely used deep learning architectures VGG16, VGG19, InceptionV3, MobileNetV2, ResNet50, and DenseNet, to systematically assess their robustness and diagnostic performance under these unconstrained conditions. Based on this comparative analysis, the most effective architecture is further optimized through targeted architectural and training enhancements, achieving improved accuracy and reliability in AMD classification. Together, these contributions address a largely overlooked challenge in ophthalmic AI: building detection systems that generalize beyond idealized imaging settings toward practical and clinically deployable solutions.

The remainder of this paper is organized as follows. Section 2 reviews the related work on AMD detection and deep learning-based medical image analysis. Section 3 describes the proposed dataset, including data acquisition, preprocessing steps, and the characteristics designed to simulate unconstrained imaging conditions. Section 4 presents the experimental setup and reports the results of the comparative evaluation of the deep learning models, followed by a detailed discussion of the findings. Finally, Section 5 concludes the paper and outlines potential directions for future research.

## 2. Related Work

The application of deep learning to retinal image analysis has garnered considerable attention in recent years, with numerous studies demonstrating strong performance in the automated detection and classification of various ocular diseases. This section reviews key contributions relevant to AMD detection, retinal disease classification, and dataset-driven model optimization.

Early work by Peng et al. [15] demonstrated the potential of deep learning for predicting the risk of late-stage AMD from color fundus photographs. Their model, trained on large-scale longitudinal data, achieved performance comparable to that of expert retinal specialists, thereby establishing a strong baseline for data-driven AMD risk stratification. Building upon this direction, Chen et al. [8] proposed a multi-modal, multi-task, multi-attention (M^3^) deep learning framework for the detection of reticular pseudodrusen, a key AMD biomarker. By leveraging complementary imaging modalities and attention mechanisms, their approach achieved robust classification across AMD subtypes, underscoring the value of multi-modal data fusion in addressing the complexity of AMD manifestations.

Several studies have focused on transfer learning with established convolutional neural network (CNN) architectures for retinal disease classification. Jain et al. [23] proposed a hybrid model combining a modified CNN with Long Short-Term Memory (LSTM) networks for retinal disease prediction, demonstrating that integrating spatial and sequential feature extraction can enhance diagnostic accuracy. Similarly, Sajid and Ibrahim [7] introduced HDR-EfficientNet, an optimized EfficientNet-based architecture tailored for the simultaneous classification of hypertensive and diabetic retinopathy. Their work highlighted the effectiveness of architecture-level optimization in improving model robustness and generalization. Rakib et al. [12] further explored EfficientNet variants for automated retinal disease classification using fundus images, reporting competitive performance across multiple disease categories and reinforcing the suitability of lightweight architectures for clinical deployment.

Beyond classification with standard architectures, recent work has explored more advanced modeling strategies. Koseoglu et al. conducted a comprehensive review of deep learning applications for AMD classification and detection using optical coherence tomography (OCT) imaging, cataloging the strengths and limitations of CNN-based approaches and identifying generalizability as a persistent challenge across existing methods [21]. Leng et al. performed a systematic review and meta-analysis of deep learning diagnostic accuracy studies for AMD detection, reporting pooled sensitivity and specificity values of 94% and 97%, respectively, while noting that dataset variability and imaging modality remain critical factors influencing model performance [17]. More recently, Tsuji et al. proposed a VGG-16-based deep learning model for classifying subtypes of neovascular AMD from OCT images, achieving 87.4% accuracy and outperforming several ophthalmologists, thereby validating the clinical utility of CNN-based diagnostic systems even under challenging multi-class settings [24].

Despite these advances, a common limitation across the reviewed studies is their reliance on datasets acquired under controlled or standardized imaging conditions, which may not reflect the variability encountered in real-world clinical environments. The present work addresses this gap by constructing a novel AMD dataset that explicitly incorporates noise, luminance variations, and device-related artifacts, while systematically evaluating multiple deep learning architectures under these unconstrained conditions.

Furthermore, the majority of existing methods report performance metrics such as accuracy, sensitivity, and specificity under balanced or near-balanced dataset conditions, which do not reflect the class distributions typically observed in clinical AMD screening. Leng et al. [17] reported pooled sensitivity and specificity values of 94% and 97%, respectively across the reviewed studies; however, their meta-analysis also acknowledged that dataset variability, imaging modality differences, and inconsistent evaluation protocols make direct cross-study comparisons unreliable. Koseoglu et al. [21] similarly identified generalizability as a persistent challenge, noting that most CNN-based AMD models are optimized for specific imaging modalities and fail to maintain performance consistency across different devices or patient populations. Rakib et al. [12] and Sajid and Ibrahim [7] demonstrated competitive results using EfficientNet-based architectures; however, both studies were conducted on datasets with homogeneous imaging characteristics, leaving the impact of real-world artifacts on model performance unaddressed. In contrast, the present work explicitly confronts these shortcomings by introducing a dataset that reflects natural clinical variability—including luminance inconsistencies, motion artifacts, and device-related noise—and by conducting a systematic comparative evaluation of six deep learning architectures under these unconstrained conditions, thereby providing a more realistic assessment of diagnostic performance in practical screening environments.

## 3. Dataset

### 3.1. Fluorescein Angiography (FFA) Imaging in Retinal Diagnostics

Fluorescein angiography (FFA) is a widely adopted diagnostic imaging technique in ophthalmology, primarily used for the evaluation of retinal and choroidal vasculature. The procedure involves the intravenous administration of sodium fluorescein dye, followed by sequential imaging to assess blood flow dynamics, detect vascular abnormalities, and monitor the progression of retinal diseases.

Prior to imaging, the patient’s pupils are dilated using mydriatic eye drops to optimize image acquisition. Intravenous access is then established for the administration of fluorescein dye, typically delivered as either 5 mL of a 10% solution or 2.5 mL of a 25% solution. The dye is injected into a peripheral vein, and imaging commences immediately to document the transit of fluorescein through the ocular circulation.

Fluorescein angiography unfolds in three distinct phases, each providing critical information about retinal and choroidal hemodynamics. The early phase begins approximately 10–15 s post-injection, capturing arterial filling and offering a detailed view of the choroidal circulation. The mid-phase follows, documenting venous filling and capillary perfusion throughout the retina, thereby providing valuable insights into overall vascular function. Finally, the late phase is characterized by the gradual dissipation of the dye, during which abnormalities such as leakage, pooling, or staining become apparent, potentially indicating underlying vascular pathology. Each phase contributes distinctly to a comprehensive assessment of retinal health.

**Figure 1:**
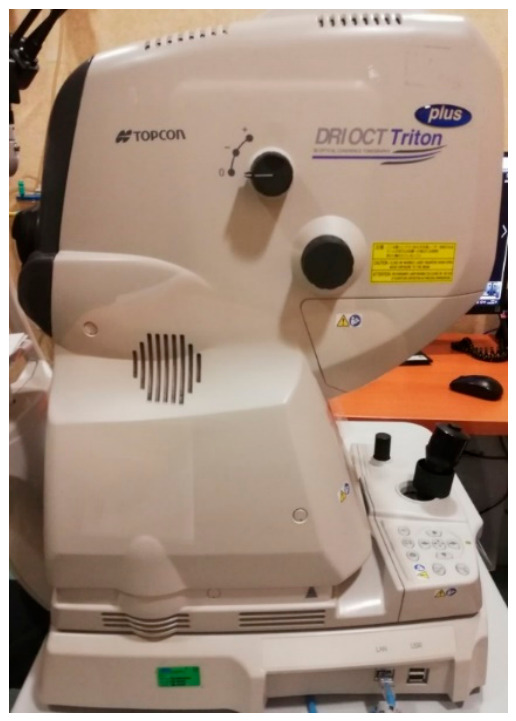
Topcon DRI Triton OCT Plus device used to gather the proposed dataset.

The acquired images are systematically analyzed to identify abnormalities including microaneurysms, capillary non-perfusion, neovascularization, vascular leakage, staining, and blockage resulting from hemorrhage or other pathological changes. Modern FFA imaging systems combine high-speed acquisition with advanced visualization capabilities to maximize diagnostic accuracy. In this study, imaging was performed using the Topcon DRI Triton OCT Plus (see Figure 1), a state-of-the-art multimodal imaging platform that offers several key features relevant to retinal diagnostics. Its widefield imaging capability enables comprehensive visualization of the retina, including peripheral regions that are critical for detecting ischemia and peripheral neovascularization. The system further provides high-resolution image quality, facilitating detailed examination of retinal and choroidal vessels and ensuring reliable detection of subtle vascular abnormalities. Importantly, the Topcon DRI Triton OCT Plus supports multimodal integration, combining FFA with structural optical coherence tomography (OCT), optical coherence tomography angiography (OCTA), and color fundus photography. This multimodal capability enables the simultaneous assessment of both vascular and structural abnormalities, substantially enhancing diagnostic insight and clinical decision-making.

Fluorescein angiography serves as an indispensable tool in the diagnosis and longitudinal monitoring of a broad spectrum of retinal diseases. It is particularly effective in identifying microaneurysms, retinal ischemia, and neovascularization associated with diabetic retinopathy. In the context of age-related macular degeneration (AMD), FFA facilitates the visualization of choroidal neovascularization and associated leakage patterns, which are hallmarks of the wet AMD subtype. For retinal vascular occlusions, it aids in delineating ischemic regions and venous irregularities, while in uveitis, it highlights vascular leakage attributable to intraocular inflammation. Furthermore, FFA findings frequently correlate with retinal thickening observed on OCT imaging, rendering it particularly valuable in the evaluation and management of macular edema. This broad diagnostic capability underscores the central role of FFA in the clinical management of retinal pathologies and motivates its use as the primary imaging modality for dataset construction in this study.

### 3.2. Data Statistics

**Figure 2:**
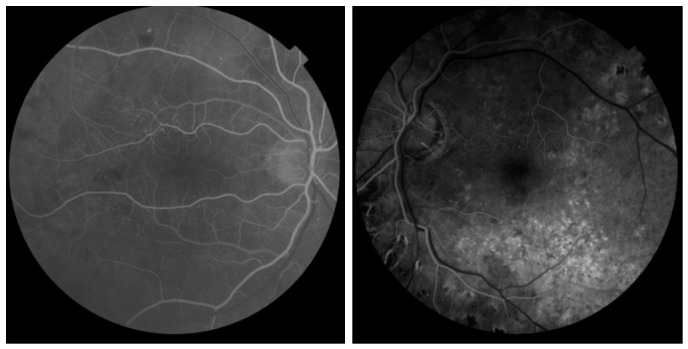
One normal (**left**) and one diseased (**right**) sample image from our dataset.

The dataset comprises a total of 1783 retinal FFA photographs collected from 163 individuals, categorized into two groups: normal eyes and AMD-affected (diseased) eyes. The photographs were distributed across right and left eyes in a roughly balanced manner, ensuring symmetric ocular representation within the dataset. All images were standardized to a uniform resolution of 1932 × 1932 pixels to maintain consistency across samples and facilitate model training. Representative examples of normal and diseased retinal FFA photographs from the dataset are illustrated in Figure 2.

Patient Inclusion Criteria: Participants were selected based on the following criteria: (1) confirmed diagnosis of AMD (dry or wet) by certified and expert ophthalmologists using standard clinical examinations and imaging findings for the diseased group; (2) absence of any retinal pathology, prior ocular surgery, or systemic conditions known to affect retinal appearance for the normal group; and (3) age of 40 years or older, consistent with the typical demographic at risk for AMD.

Annotation Process: All retinal photographs were annotated by two experienced ophthalmologists, each with a minimum of 5 years of clinical experience in retinal disease diagnosis. Each image was independently labeled as either normal or AMD-affected. In cases of disagreement between the two annotators, more analysis was conducted to decide, and the image was deleted from the dataset in cases of uncertainty.

Dataset Accessibility: To support reproducibility and facilitate further research in AMD detection, the dataset is intended to be made publicly available upon acceptance of this manuscript. Access will be provided through GitHub, subject to a data-sharing agreement that ensures patient anonymity and compliance with relevant ethical and data protection regulations.

The dataset exhibits a degree of class imbalance, with diseased cases outnumbering normal cases, reflecting the real-world clinical distribution commonly observed in AMD screening scenarios. A detailed summary of the dataset composition is provided below (see Table 1).

- Normal Eyes: A total of 626 photographs were obtained from 75 healthy individuals. Most participants contributed an average of 8–9 images, while a subset provided only a single photograph, reflecting variability in clinical data availability.
- Diseased Eyes: A total of 1157 photographs were collected from 88 individuals diagnosed with AMD. The number of images per participant ranged from 12 to 14, encompassing both dry and wet AMD conditions and thereby capturing the full spectrum of disease manifestations.

In our dataset, the term “unconstrained” refers to the natural, real-world variability inherent in the dataset, which was collected from routine clinical screening sessions without any standardization of imaging conditions or acquisition protocols. Unlike curated benchmark datasets, where imaging parameters are carefully controlled, the images in this dataset were acquired across different clinical sessions, operators, and device settings, resulting in a diverse range of naturally occurring artifacts and variations. These include:

- Luminance and exposure variations: arising from differences in flash intensity, patient pupil dilation levels, and ambient lighting conditions during acquisition.
- Noise and graininess: resulting from low-quality dye injection timing, patient movement, or suboptimal camera focus during fluorescein angiography.
- Contrast inconsistencies: stemming from variability in fluorescein dye concentration, injection timing, and individual patient vascular response.
- Blur and motion artifacts: caused by involuntary patient eye movement or blinking during image capture.
- Device-related inconsistencies: reflecting the use of different fundus camera models and settings across clinical sessions.

Importantly, all variations present in the dataset are entirely natural in origin; no synthetic augmentation or artificial artifact injection was applied during data collection. This naturalistic variability is precisely what distinguishes the proposed dataset from existing controlled benchmarks and constitutes the core of its “unconstrained” characterization.

## 4. Deep Learning Architectures and Experimental Setup

This section describes the deep learning architectures employed in this study, along with the experimental configurations and optimization strategies adopted for AMD detection using the constructed dataset. Six well-established CNN architectures were evaluated in a comparative study, followed by targeted optimization of the best-performing model to maximize classification performance.

### 4.1. Compared Architectures

Six widely adopted deep learning architectures were selected for evaluation: VGG16, VGG19, InceptionV3, MobileNetV2, ResNet50, and DenseNet. A brief description of each architecture is provided below:

VGG16 is a convolutional neural network introduced by Simonyan and Zisserman for large-scale image recognition. The architecture employs small 3 × 3 convolutional filters across 16 weight layers, enabling effective hierarchical feature extraction. Its sequential and uniform design facilitates straightforward implementation; however, its relatively large parameter counts results in higher computational demands [1].

VGG19 extends the VGG16 architecture by incorporating three additional convolutional layers, bringing the total depth to 19 weight layers. Like its predecessor, VGG19 utilizes 3 × 3 convolutional filters, allowing it to capture complex spatial patterns while maintaining architectural simplicity. The added depth is intended to improve feature representation, though it also increases computational cost [1].

InceptionV3, developed by Szegedy et al. as part of the GoogLeNet family, introduces the concept of inception modules that perform parallel convolutional operations with varying kernel sizes. This design enables efficient multi-scale feature extraction within a single layer, making InceptionV3 well-suited for analyzing complex retinal structures such as those associated with AMD [2].

MobileNetV2, introduced by Sandler et al., is specifically designed for resource-constrained environments such as mobile and edge devices. It employs depthwise separable convolutions combined with inverted residual blocks and linear bottlenecks, substantially reducing computational overhead while preserving competitive classification performance. These properties make it a practical candidate for scalable AMD detection systems [5].

ResNet50, proposed by He et al., is a 50-layer deep residual network that addresses the vanishing gradient problem through the introduction of skip connections. These residual connections enable the network to learn deeper representations without performance degradation, making ResNet50 particularly effective for feature extraction in medical imaging tasks [6].

DenseNet, introduced in [25], employs a densely connected architecture in which each layer receives feature maps from all preceding layers. This design promotes maximum information and gradient flow throughout the network, resulting in improved parameter efficiency and feature reuse. DenseNet is especially well-suited for detecting subtle pathological patterns in medical imaging datasets, and its compact design helps mitigate overfitting, particularly when training data are limited.

### 4.2. Experimental Configuration

In the comparative experiment, the dataset was partitioned into training and testing subsets, and all six architectures were applied to the binary classification task of AMD detection. Table 2 summarizes the architectural configurations and training hyperparameters adopted for each model.

All models were trained using the Adam optimizer with a learning rate of 0.0001, with the exception of ResNet50, which employed the AdamW optimizer. Transfer learning was applied where indicated, with initial weights pre-trained on ImageNet to leverage generalizable low-level feature representations. Data augmentation strategies—including horizontal flipping, random rotation, width and height shifts, shearing, zooming, and brightness adjustment—were selectively applied to improve model generalization and reduce overfitting, particularly for models with larger parameter spaces. A batch size of 32 was used across most configurations, with the exception of VGG19, which employed a batch size of 16.

### 4.3. Optimization of the VGG16 Model

Given that VGG16 demonstrated the strongest baseline performance in the comparative experiment, a systematic optimization study was conducted to further enhance its classification capability for AMD detection. Although the baseline VGG16 configuration achieved competitive accuracy, several architectural and training-level modifications were explored to improve its sensitivity, generalization, and overall robustness for this specific clinical task.

The optimization process investigated modifications across three main directions. First, the depth of the classification head was varied by experimenting with different numbers of dense layers—ranging from one to four—to assess the impact of classifier capacity on overall performance. Second, regularization strategies were examined, including the incorporation of dropout layers between dense layers to mitigate overfitting and batch normalization layers to stabilize training dynamics and accelerate convergence. Third, various training configurations were explored, including different epoch counts, to identify the most effective trade-off between model convergence and generalization.

Throughout all experiments, the 13 convolutional layers of the pre-trained VGG16 model were frozen to preserve the generalizable low-level and mid-level feature representations learned from ImageNet, thereby preventing overfitting on the relatively small medical imaging dataset. The Adam optimizer with a learning rate of 0.0001 was adopted as the primary optimization algorithm, and data augmentation techniques—specifically horizontal flipping and random image rotation (range: 20°)—were applied to further enhance model generalization. A batch size of 32 was maintained consistently across all optimization experiments. The results of all explored configurations and their corresponding performance metrics are presented and analyzed in Section 5.2.

## 5. Results and Discussion

### 5.1. Comparative Performance of CNN Architectures

Table 3 presents the classification performance of all six CNN architectures evaluated for the AMD detection task. The results reveal notable variations across models, reflecting differences in architectural design, training configuration, and capacity for feature extraction.

Among all evaluated models, VGG16 achieved the highest overall accuracy of 86%, demonstrating strong feature extraction and classification capabilities for the AMD detection task. However, accuracy alone is insufficient for a comprehensive evaluation in clinical contexts, and additional metrics such as precision, recall, and F1-score provide deeper insight into each model’s diagnostic behavior.

A notable trade-off between precision and recall is observed across the compared architectures. InceptionV3 and MobileNetV2 exhibit the highest recall values of 95% and 91%, respectively, indicating strong sensitivity to AMD-positive cases. This characteristic renders them particularly suitable for screening applications where minimizing false negatives (that is, missed diagnoses) is of paramount clinical importance. In contrast, VGG16 achieves a more balanced distribution between precision (78%) and recall (82%), resulting in a stable F1-score of 80%. This balance suggests greater robustness in simultaneously reducing both false positives and false negatives, which is critical in clinical decision-support systems where both error types carry significant consequences.

**Figure 3:**
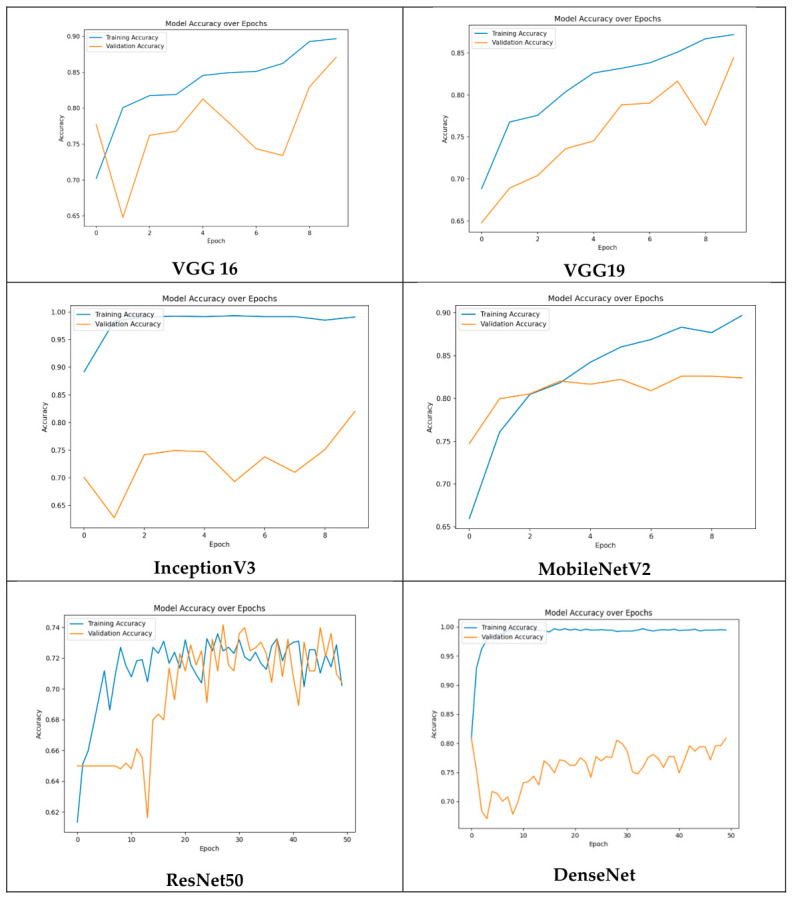
Training and validation accuracy curves for each evaluated architecture.

ResNet50 and DenseNet were trained for 50 epochs, yet neither surpassed the performance of models trained for only 10 epochs. While DenseNet achieved an acceptable F1-score of 76%, ResNet50 recorded the lowest scores across most metrics despite prolonged training. This outcome may reflect optimization challenges inherent to deeper architectures when applied to relatively small, domain-specific datasets and suggests that additional fine-tuning or architectural adaptation may be required to unlock their full potential. The training and validation accuracy curves for all models are illustrated in Figure 3, providing further insight into convergence behavior and potential overfitting across architectures.

From a clinical deployment perspective, models with high recall, such as InceptionV3 and MobileNetV2, may be preferable in population-level AMD screening programs where sensitivity is prioritized. However, VGG16’s balanced performance across all metrics makes it a more reliable choice for general clinical deployment scenarios. These findings motivated the selection of VGG16 as the architecture for further optimization, as detailed in Section 4.3.

### 5.2. Performance of the Optimized VGG16 Model

This subsection presents and analyzes the results of the systematic optimization experiments conducted on the VGG16 architecture, as described in Section 4.3. Table 4 summarizes the performance metrics obtained across all explored architectural configurations and training settings.

The results reveal several important trends regarding the impact of architectural complexity and regularization on classification performance. With respect to classifier depth, increasing the number of dense layers beyond one consistently led to performance degradation. The two-dense-layer configuration achieved 85% accuracy, while the three-dense-layer variant further declined to 79% accuracy, with a notably low recall of 56%, suggesting that excessive classifier depth introduces optimization difficulties and hinders the model’s ability to generalize to AMD-positive cases. The four-dense-layer configuration trained for 10 epochs achieved a high recall of 96%, but at the cost of reduced precision (70%), indicating a tendency toward over-prediction of the positive class.

Regarding regularization, the incorporation of dropout layers between dense layers improved recall considerably, reaching up to 96% but consistently reduced precision, reflecting a shift in the model’s decision boundary toward greater sensitivity at the expense of specificity. The addition of batch normalization layers alongside dropout resulted in a substantial collapse in precision (48%), despite achieving perfect recall of 100%. This behavior suggests that the combined effect of dropout and batch normalization caused the model to default toward predicting the majority class, rendering the configuration unreliable for balanced clinical decision-making.

Extended training across configurations generally did not yield consistent improvements. In several cases, increasing the number of epochs led to reduced accuracy and F1-score, indicating overfitting despite the relatively small size of the classification head. This further confirms the importance of early stopping and careful epoch selection in optimizing models for small medical imaging datasets.

The optimal configuration was identified as comprising 13 convolutional layers, 5 max pooling layers, 1 global average pooling layer, and a single dense layer for binary classification, trained for 13 epochs. This configuration achieved the best overall performance, with an accuracy of 88%, precision of 81%, recall of 83%, and an F1-score of 83%. These results represent a meaningful improvement over the baseline VGG16 configuration reported in Table 3 and confirm that preserving the pre-trained convolutional feature representations while minimizing classifier complexity is the most effective strategy for this dataset. The findings demonstrate that architectural simplicity, combined with appropriate transfer learning and targeted data augmentation, yields superior and more balanced classification performance for AMD detection under unconstrained imaging conditions.

### 5.3. Limitations and Failure Case Analysis

**Figure 4:**
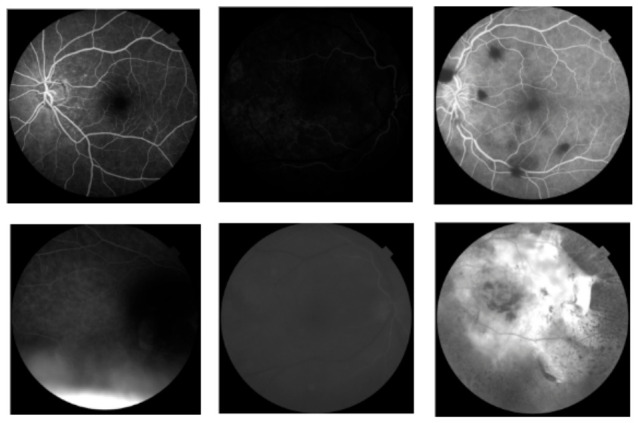
Representative examples of retinal images misclassified by the optimized model.

Despite the improved performance of the optimized VGG16 model, certain limitations remain, particularly in handling retinal images of suboptimal quality. As illustrated in Figure 4, the model struggles to correctly classify images that are excessively dim, as insufficient contrast obscures the fine-grained features necessary for reliable classification. Similarly, structurally normal retinal images containing dark peripheral regions are occasionally misclassified as AMD-positive, as the model may erroneously interpret these areas as pathological findings. Overexposed images with abnormally high brightness, as well as blurry images lacking sufficient structural detail, also pose significant challenges, as they fail to provide the visual cues required for effective feature extraction.

These failure cases highlight the model’s sensitivity to image quality and its dependence on well-defined visual input. Importantly, however, these challenging cases also serve a constructive role in simulating the variability inherent to real-world clinical imaging environments. In practice, retinal images may vary considerably in brightness, contrast, and sharpness due to factors such as patient movement, device calibration inconsistencies, or suboptimal acquisition conditions. By incorporating such cases into the dataset, this study establishes a realistic and challenging benchmark that more faithfully reflects operational clinical conditions. These limitations identify clear directions for future research, including the integration of advanced preprocessing pipelines, domain adaptation techniques, noise-robust model architectures, and multimodal data fusion strategies. Addressing these challenges will be essential for developing AMD detection systems that are both clinically reliable and deployable across diverse imaging environments.

Another limitation of the current study is the absence of a formal quantitative inter-observer agreement analysis. While all retinal images were independently reviewed and labeled by two experienced ophthalmologists—with disagreements resolved through consensus by a third senior specialist—a formal statistical measure of inter-observer agreement, such as Cohen’s Kappa coefficient, was not computed during the annotation process. This was primarily due to the retrospective nature of the data collection, where annotations were performed as part of routine clinical practice rather than within a structured research annotation protocol. We acknowledge that quantitative inter-observer agreement is an important indicator of label reliability and dataset quality, and the absence of this metric is recognized as a limitation of the present work. Future dataset collection efforts will incorporate a formally designed annotation protocol that includes independent labeling by multiple annotators and systematic computation of inter-observer agreement statistics to ensure the highest standards of annotation reliability and scientific rigor.

Despite the promising results achieved by the proposed deep learning framework, it is important to acknowledge the inherent limitations of automated diagnostic systems and their current role within clinical practice. Deep learning models, while capable of achieving high classification accuracy, remain fundamentally dependent on the quality, diversity, and representativeness of the training data. Models trained on a specific population or imaging device may exhibit performance degradation when deployed in different clinical environments, a challenge that persists across the broader field of medical AI. Furthermore, deep learning systems lack the contextual clinical reasoning that experienced ophthalmologists bring to diagnosis, including the integration of patient history, systemic comorbidities, and subtle morphological nuances that may not be fully captured through image-based classification alone. The question of whether such systems can completely replace clinicians is therefore not merely a technical one, but also an ethical and regulatory consideration. At present, the proposed framework is best positioned as a clinical decision-support tool, intended to assist rather than replace ophthalmologists by providing a fast, consistent, and scalable first-line screening mechanism, particularly in resource-limited settings where access to retinal specialists is constrained. Human expert oversight remains essential for final diagnosis, treatment planning, and patient communication, and the integration of AI-assisted tools into clinical workflows should be approached with appropriate validation, transparency, and regulatory compliance.

## 6. Conclusions

This paper presented a comprehensive study on the automated detection of age-related macular degeneration (AMD) from retinal fluorescein angiography (FFA) images acquired under unconstrained clinical conditions. To address the limitations of existing approaches that rely predominantly on datasets collected under controlled imaging environments, a novel AMD dataset was constructed comprising 1783 retinal photographs from 163 individuals, incorporating real-world imaging challenges such as noise, luminance variations, and device-related artifacts. This dataset provides a more realistic and clinically representative benchmark for evaluating the robustness of deep learning-based diagnostic systems.

A systematic comparative evaluation of six widely adopted CNN architectures: VGG16, VGG19, InceptionV3, MobileNetV2, ResNet50, and DenseNet121 was conducted to assess their suitability for AMD binary classification. The experimental results demonstrated notable performance variations across architectures, highlighting the significant influence of architectural design, training configuration, and dataset characteristics on model robustness. Among the evaluated models, VGG16 achieved the highest baseline accuracy of 86%, along with a well-balanced precision–recall trade-off, making it the most suitable candidate for further optimization.

Through a systematic optimization study involving architectural modifications, regularization strategies, and targeted training configurations, the VGG16 model was further refined to achieve an accuracy of 88%, a precision of 81%, a recall of 83%, and an F1-score of 83%. These results confirm that architectural simplicity, combined with transfer learning from ImageNet pre-trained weights and selective data augmentation, constitutes an effective strategy for AMD detection under challenging imaging conditions. The findings further demonstrate that increasing classifier depth and introducing excessive regularization do not necessarily improve performance and may, in fact, hinder generalization on small, domain-specific medical imaging datasets.

Despite these promising results, the study identified several limitations related to image quality, including the misclassification of excessively dim, overexposed, and blurry retinal images. These failure cases, while reflecting the current boundaries of the proposed model, simultaneously underscore the clinical relevance of the constructed dataset as a challenging and realistic benchmark. Addressing these limitations represents an important direction for future research, including the development of advanced preprocessing pipelines, noise-robust architectures, and domain adaptation techniques to improve model performance under diverse imaging conditions.

Future work will pursue several directions to further advance the proposed framework. These include the integration of multimodal imaging data such as OCT and OCTA alongside FFA images to provide complementary structural and vascular information for more accurate AMD characterization. More recent architectures, including Vision Transformers and hybrid CNN-Transformer models, will also be explored to enhance classification performance and generalizability. Dataset expansion to include a larger and more diverse patient population will be prioritized to improve statistical reliability and clinical applicability. Furthermore, a more rigorous evaluation will be conducted through validation on additional publicly available AMD datasets, inclusion of confusion matrices and ROC/AUC analyses, and integration of explainability methods such as Grad-CAM to improve the clinical interpretability and trustworthiness of the proposed system.

In summary, this study contributes a clinically realistic AMD dataset and demonstrates the effectiveness of an optimized VGG16-based deep learning framework for robust AMD detection under unconstrained imaging conditions. The findings provide a valuable foundation for the development of reliable, scalable, and clinically deployable automated diagnostic tools for age-related macular degeneration.

## Figures and Tables

**Table 1:** Dataset Summary.

Parameter	Normal Eyes	Diseased Eyes
Number of Photographs	626	1157
Number of Individuals	75	88
Average Photos per Individual	8–9	12–14
Image Dimensions	1932 × 1932 pixels	1932 × 1932 pixels
Condition Type	N/A (Healthy)	Both Dry and Wet Cases

**Table 2:** Architectural configurations and training settings for each CNN model.

Model	Architecture	Optimizer	Learning Rate	Augmentation	Batch Size
VGG16	13 Conv, 5 MaxPool, 1 GAP, 2 Dense	Adam	0.0001	Flip, Rotation (20°)	32
VGG19	16 Conv, 5 MaxPool, 1 GAP, 1 Dense	Adam	0.0001	Flip, Rotation (20°)	16
InceptionV3	94 Conv, 4 MaxPool, 9 AvgPool, 1 GAP, 1 Dense	Adam	0.0001	None	32
MobileNetV2	35 Conv, 17 DepthwiseConv, 1 GAP, 1 Dense	Adam	0.0001	Flip, Rotation, Shift, Shear, Zoom, Brightness	32
ResNet50	49 Conv, 1 Dense	AdamW	0.0001	None	32
DenseNet121	428 Conv, 1 GAP, 1 Dense	Adam	0.00005	None	32

**Table 3:** Performance comparison of CNN models for AMD detection.

Name	Epochs	Accuracy	Precision	Recall	F1-Score
VGG16	10	86%	78%	82%	80%
VGG19	10	83%	72%	83%	77%
InceptionV3	10	82%	67%	95%	79%
MobileNetV2	10	82%	69%	91%	78%
Resnet50	50	75%	63%	71%	67%
DenseNet	50	80%	67%	88%	76%

**Table 4:** Experimental results of performance optimization techniques applied to the VGG16 architecture.

Architecture	Epochs	Accuracy	Precision	Recall	F1-Score
13 Conv, 5 MaxPool, 1 GAP, 1 Dense	13	88%	81%	83%	83%
13 Conv, 5 MaxPool, 1 GAP, 2 Dense	20	85%	79%	80%	79%
13 Conv, 5 MaxPool, 1 GAP, 3 Dense	30	79%	77%	56%	65%
13 Conv, 5 MaxPool, 1 GAP, 4 Dense	10	84%	70%	96%	81%
13 Conv, 5 MaxPool, 1 GAP, 4 Dense	20	77%	63%	85%	72%
13 Conv, 5 MaxPool, 1 GAP, 3 Dense, 2 Dropout	10	81%	66%	95%	78%
13 Conv, 5 MaxPool, 1 GAP, 3 Dense, 2 Dropout	20	79%	64%	96%	76%
13 Conv, 5 MaxPool, 1 GAP, 3 Dense, 2 Dropout, 2 BN	10	62%	48%	100%	65%
13 Conv, 5 MaxPool, 1 GAP, 4 Dense, 3 Dropout	10	65%	70%	70%	70%

## Data Availability

The data presented in this study are available on request from the corresponding author. The data are not publicly available due to privacy and ethical restrictions related to patient information.
